# Pulsed Electrolysis with a Nickel Molecular Catalyst Improves Selectivity for Carbon Dioxide Reduction

**DOI:** 10.1021/jacs.3c04811

**Published:** 2023-07-05

**Authors:** Francesca Greenwell, Bhavin Siritanaratkul, Preetam K. Sharma, Eileen H. Yu, Alexander J. Cowan

**Affiliations:** † Department of Chemistry and Stephenson Institute for Renewable Energy, University of Liverpool, Liverpool L69 7ZF, United Kingdom; ‡ Department of Chemical Engineering, Loughborough University, Loughborough LE11 3TU, United Kingdom

## Abstract

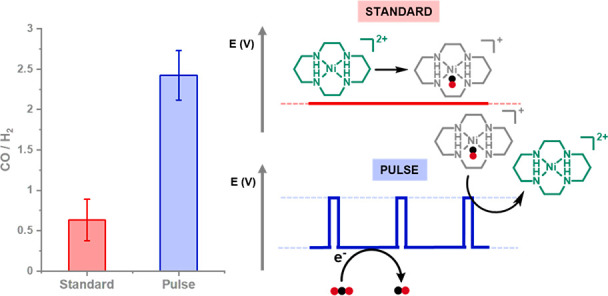

Pulsed electrolysis can significantly improve carbon dioxide reduction on metal electrodes, but the effect of short (millisecond to seconds) voltage steps on molecular electrocatalysts is largely unstudied. In this work, we investigate the effect pulse electrolysis has on the selectivity and stability of the homogeneous electrocatalyst [Ni(cyclam)]^2+^ at a carbon electrode. By tuning the potential and pulse duration, we achieve a significant improvement in CO Faradaic efficiencies (85%) after 3 h, double that of the system under potentiostatic conditions. The improved activity is due to in situ catalyst regeneration from an intermediate that occurs as part of the catalyst’s degradation pathway. This study demonstrates the wider opportunity to apply pulsed electrolysis to molecular electrocatalysts to control activity and improve selectivity.

Electrochemical carbon dioxide reduction (CO_2_R) holds the potential to convert CO_2_ to fuels and chemical feedstocks utilizing renewable energy. Efforts are focused on developing new electrocatalysts and controlling the electrode–electrolyte interface with existing catalysts to understand and improve their catalytic behavior.^1−4^ Experiments are typically carried out under potentiostatic or galvanostatic conditions. However, recent studies on metal electrodes have utilized pulsed electrolysis as a way to influence and improve reaction selectivity and stability in electrochemical CO_2_R.^5,6^ There are multiple proposed effects of using a pulsed voltage depending on the system and pulse parameters used, including inhibiting catalyst poisoning,^7−11^ surface oxidation, or roughening;^7,8,11−14^ rearrangement of surface coverage;^13,15−17^ and altering the local pH and CO_2_ concentration at the electrode.^18−21^

While there are many pulsed studies on different metal electrodes for CO_2_R, we are not aware of any where the impact of short (ms to s) voltage pulses is examined with homogeneous molecular catalysts despite it offering a potential way to modify catalytic activity and stability. In this work, we report a pulse electrolysis study on a homogeneous molecular catalyst for CO_2_R with an inert glassy carbon working electrode (GCE). Nickel cyclams (cyclam = 1,4,8,11-tetraazacyclotetradecane) are both well studied photo-^22−24^ and electrocatalysts for CO production in aqueous electrolytes.^25−32^ Recently, [Ni(cyclam)]^2+^ has also been found to be selective for CO production when used on gas diffusion electrodes in H cells^33,34^ and higher current density electrolyzers,^30,35^ notably even at low pH,^35^ which has made efforts toward improving its activity and stability of particular interest.

Early experiments with [Ni(cyclam)]^2+^ were carried out with the catalyst adsorbed onto Hg electrodes, but more recently, it has been shown that [Ni(cyclam)]^2+^ can also be used with a GCE.^29,36^ Here, Faradaic efficiencies (FEs) are typically lower, but the catalyst does not adsorb (Figure S2), thereby creating a simpler molecular system to study the effects of pulsed electrolysis. Figure 1A shows a cyclic voltammogram (CV) of 1 mM [Ni(cyclam)]^2+^ in 0.5 M NaCl using a GCE. Under Ar, the CV remains fairly featureless as hydrogen evolution obscures the Ni(II)/(I) couple in aqueous electrolyte.^29^ Under CO_2_, we see a significant increase in current at −1.5 V versus Ag/AgCl, thereby indicating CO_2_R and the appearance of two small anodic features at −1.3 V (i) and −0.5 V (ii), which are assigned to the oxidation of deactivated catalyst species [Ni(cyclam)(CO)]^+^ and further irreversibly reduced Ni(0) carbonyl, respectively.^37^ The formation of [Ni(cyclam)(CO)]^+^ as a result of the high CO binding constant to [Ni(cyclam)]^+^ (*K*_CO_ = 7.5 × 10^5^, *K*_CO2_ = 16) has been proposed to be the cause of the low stability and selectivity of the catalyst when used at both GCE and gas diffusion electrodes.^33,35,37^ More widely, CO poisoning and overreduction of intermediates has been proposed to limit stable electrochemical CO_2_R in a range of molecular catalysts, with metal centers including Ni,^38,39^ Fe,^40,41^ Co, etc.^42−44^ Remediation methods have included the removal of CO with scavengers or increased gas flow,^35,37^ modifications to the catalyst structure,^40−44^ or instating long periods (minutes to hours) for recovery/regeneration, which only leads to a temporary recovery in activity.^33,35^

**Figure 1:**
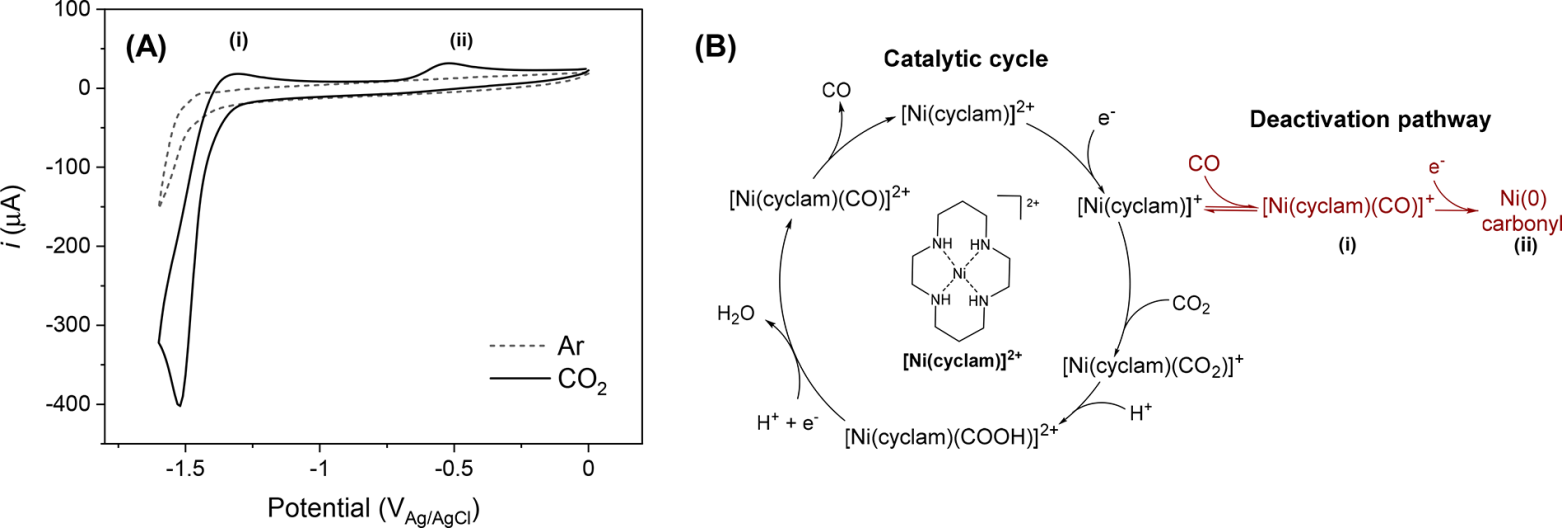
(A) CV of 1 mM [Ni(cyclam)]^2+^ in 0.5 M NaCl at a GCE at 1 V/s (0 to −1.6 to 0 V) under Ar and CO_2_ versus Ag/AgCl, Pt counter separated by vycor frit, recorded without *iR* compensation. Plotted using IUPAC convention. (B) Reported catalytic cycle and deactivation pathway of [Ni(cyclam)]^2+^.^29^

In this work, we incorporate a short 40 ms to 1 s asymmetric anodic pulse (*E*_A_) throughout electrolysis to enable stable operation (see Tables S1–3 and Figures S3–7 for chronoamperometry data). Figure 2 shows potentiostatic (denoted as Standard) and pulsed electrolysis experiments of 0.1 mM Ni(cyclam) in CO_2_-saturated 0.5 M NaCl over 3 h at a GCE. The standard electrolysis was held at a cathodic potential (*E*_C_) of −1.6 V versus Ag/AgCl throughout. For initial pulsed electrolysis studies, *E*_C_ was held for 5 s (*t*_C_) before an anodic potential (*E*_A_) of −1.0 V versus Ag/AgCl was applied for 0.2 s (*t*_A_). The pulsed voltage profile led to a 4-fold increase in selectivity for CO (CO/H_2_ = 2.42 ± 0.10), which was stable over 3 h, compared with the standard run (CO/H_2_ = 0.63 ± 0.21). The pH of the electrolyte was measured before and after electrolysis, where a slight increase was observed postelectrolysis [from 6.3 to 7.8 (standard), 7.7 (pulsed)] in both the standard and pulsed run. Table S4 shows that the total cell energy efficiency for CO_2_ to CO of the pulse system is almost double that of the standard experiment, thereby demonstrating that any energy losses associated with the voltage pulse are offset by the increased FE for CO and higher CO production rate.

**Figure 2:**
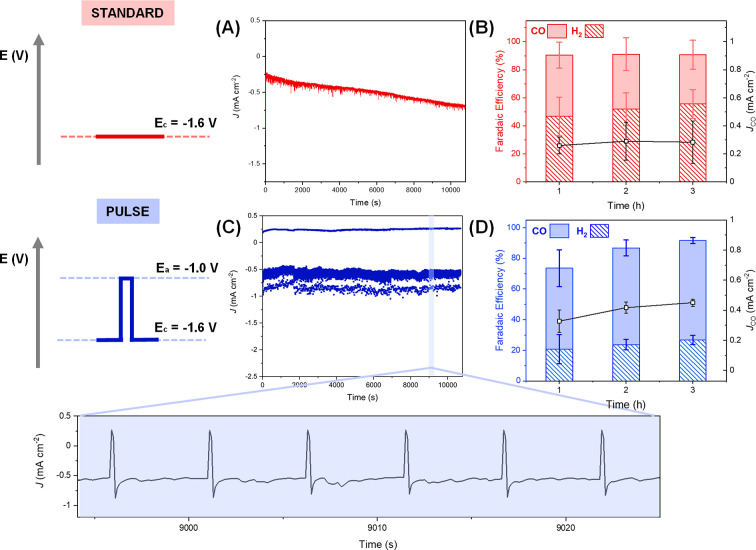
Comparison of standard (*E*_C_ = −1.6 V_Ag/AgCl_) and pulsed (*E*_C_ [*t*_C_] = −1.6 V_Ag/AgCl_ [5 s]; *E*_A_ [*t*_A_] = −1.0 V_Ag/AgCl_ [0.2 s] electrolysis of 0.1 mM [Ni(cyclam)]^2+^ in 0.5 M NaCl (aq) over 3 h. (A) Chronoamperometry trace of standard run, (B) FEs and CO partial current densities of standard run, (C) chronoamperometry trace of pulse run, and (D) FEs and CO partial current densities of pulse run. A kinetic analysis of the fast response of the system upon pulsing can be found alongside Figure S8.

Figure 2A and Figure S9 show that the overall current density increases during a standard electrolysis experiment and that this is due to increased hydrogen evolution. X-ray Photoelectron Spectroscopy (XPS) of rinsed GCEs after 3 h of either standard or pulse electrolysis shows Ni on the GCE poststandard electrolysis but not on the pulse electrolysis sample (Figures S10–12). The Ni XPS of the GCE poststandard electrolysis does not match that of a powder sample of [Ni(cyclam)]^2+^, and an analysis of the Ni/N peak ratio from the survey scan (0.24 poststandard electrolysis GCE and 2.36 [Ni(cyclam)]^2+^) shows that most of the deposited Ni is no longer coordinated to the cyclam ligand. Instead, we assigned it to mainly Ni(OH)_2_ (see the Supporting Information for details).^45,46^ Cyclam loss is proposed to occur following the reduction of [Ni(cyclam)(CO)]^+^ to form Ni(0) carbonyl compounds, which may oxidize upon exposure to atmosphere.^38^ An increase in hydrogen evolution following Ni(0) deposition is in line with other studies on Ni-based electrocatalysts,^37,38,47^ and here, we find that the GCE poststandard electrolysis has a decreased onset potential for hydrogen evolution when used in a fresh NaCl electrolyte (Figure S14).

The postelectrolysis XPS analysis indicates that the prevention of [Ni(cyclam)]^2+^ decomposition and subsequent Ni deposition is the reason for the increased CO_2_R selectivity upon pulsing. Pulsed electrolysis may prevent Ni deposition and prevent hydrogen evolution via both Faradaic and non-Faradaic mechanisms. We first look to non-Faradaic processes and consider if the voltage step could be leading to a rearrangement of the electrolyte, which would refresh the catalyst/CO_2_ at the GCE surface and remove species, such as [Ni(cyclam)(CO)]^+^, prior to their irreversible reduction. The largest rearrangement of the electrolyte would be expected to occur if the potential was stepped across the potential of zero charge (pzc) of the GCE. Differential capacitance measurements establish the pzc to be +0.4 V versus Ag/AgCl, which is in line with other reports (Figure S15). The pzc is significantly positive of the values of *E*_A_ (−0.3 to −1.0 V) where we see a beneficial effect of pulsing (Figure 3 and Tables S1–3).^48,49^ Some changes in the differential capacitance do occur between −0.3 and −1.0 V, but it is notable that the selectivity for CO production is approximately constant when *E*_A_ is varied between these voltages (CO/H_2_ = ∼2.0 to 2.5, Figure 3); therefore, the lack of Ni deposition and increase in selectivity for the CO_2_RR is unlikely to be caused by double layer rearrangement.

**Figure 3:**
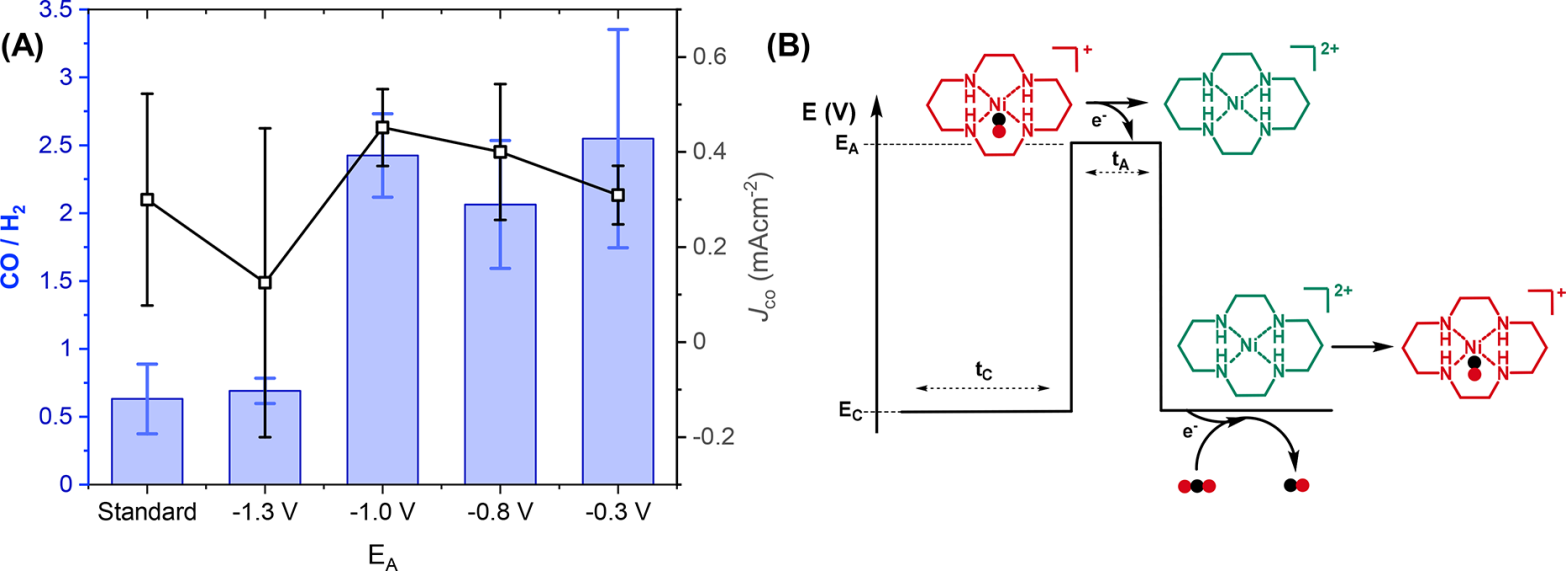
(A) Comparison of CO/H_2_ and CO partial current densities after 3 h electrolysis of 0.1 mM [Ni(cyclam)]^2+^ in 0.5 M NaCl (aq) where *E*_C_ [*t*_C_] = −1.6 V_Ag/AgCl_ [5 s]; *t*_A_= 0.2 s with changing *E*_A_. (B) Schematic illustrating the proposed mechanism of how pulsed electrolysis can reduce catalyst degradation.

We next consider whether a Faradaic process is occurring during pulsed electrolysis. Figure 3 shows that when *E*_A_ is positive of the oxidation of the Ni(0) species (−0.55 V, Figure 1), no increase in selectivity for CO_2_RR is observed when compared with experiments with *E*_A_ at −1.0 V, which suggests that Ni(0) reoxidation is not a significant pathway. When *E*_A_ = −1.3 V, the CO/H_2_ drops to 0.7 ± 0.1, which is equal to that measured under standard electrolysis conditions. The selectivity for CO is greater for pulsed runs with *E*_A_ > −1.3 V, but *J*_CO_ remains the same within error. This is because of an overall increase in current associated with increased hydrogen evolution when *E*_A_ = −1.3 V (or when nonpulsed conditions are used). The oxidation at −1.35 V in Figure 1 is assigned to [Ni(cyclam)(CO)]^+^ or [Ni(cyclam)]^2+^.^37^ Therefore, we conclude that pulsing decreases the concentration of [Ni(cyclam)(CO)]^+^ at the electrode surface, thereby preventing subsequent reduction to Ni(0) (Figure 3b). One past study employed a prolonged (10 min) oxidation of a cyclam complex at very positive potentials (+0.8 V vs RHE, approximately +0.2 V vs Ag/AgCl). This led to a short-lived recovery in the rate of CO production (∼20 min), but there was no significant decrease in hydrogen evolution, thereby demonstrating the importance of continuous removal of [Ni(cyclam)(CO)]^+^ using the pulsed voltage profile.^33^ The sensitivity of Ni(cyclam) to short/pulsed changes in applied potential may offer insight into the wide range of selectivities and stabilities when used in photocatalytic systems.^22−24^

Finally, we studied the time dependence of the anodic (*t*_A_) pulse duration while keeping *t*_C_ constant at 5 s (Figure 4). It is desirable to minimize *t*_A_ to increase the duty cycle (percentage of time that the device is held at the operating potential). The shortest *t*_A_ value we could achieve during a prolonged electrolysis experiment with our apparatus was 40 ms corresponding to a duty cycle of >99% (Table S5). Even with this very short pulse duration, we see an increase in selectivity (CO/H_2_ = 1.86 ± 0.16) when compared with the potentiostatic experiment. Analysis of the time–current response (Figure S8) indicates that ∼10 ms after the start of the anodic pulse the capacitive charging current has largely decayed and that the Faradaic current dominates. At 200 ms, there is still a significant anodic current supporting our conclusion that the increased selectivity is the result of a Faradaic process. In line with this, extension of *t*_A_ to 1 s leads to a small but measurable increase in selectivity compared with when shorter pulses are used (CO/H_2_ = 3.62 ± 0.87, FE_CO_ = ∼80%; Tables S2–4, Figure 4). However, it is important to note that the cathodic charge fraction (*Q*_C_), previously proposed to be a useful parameter for assessing pulse profiles during CO_2_R at metals,^14^ shows a large decrease when *t*_A_ = 1.0 s (*Q*_C_ = 91.2%, 96.3%, and 97.5% for *t*_A_ = 1.0, 0.2, and 0.04 s; Table S5).

**Figure 4:**
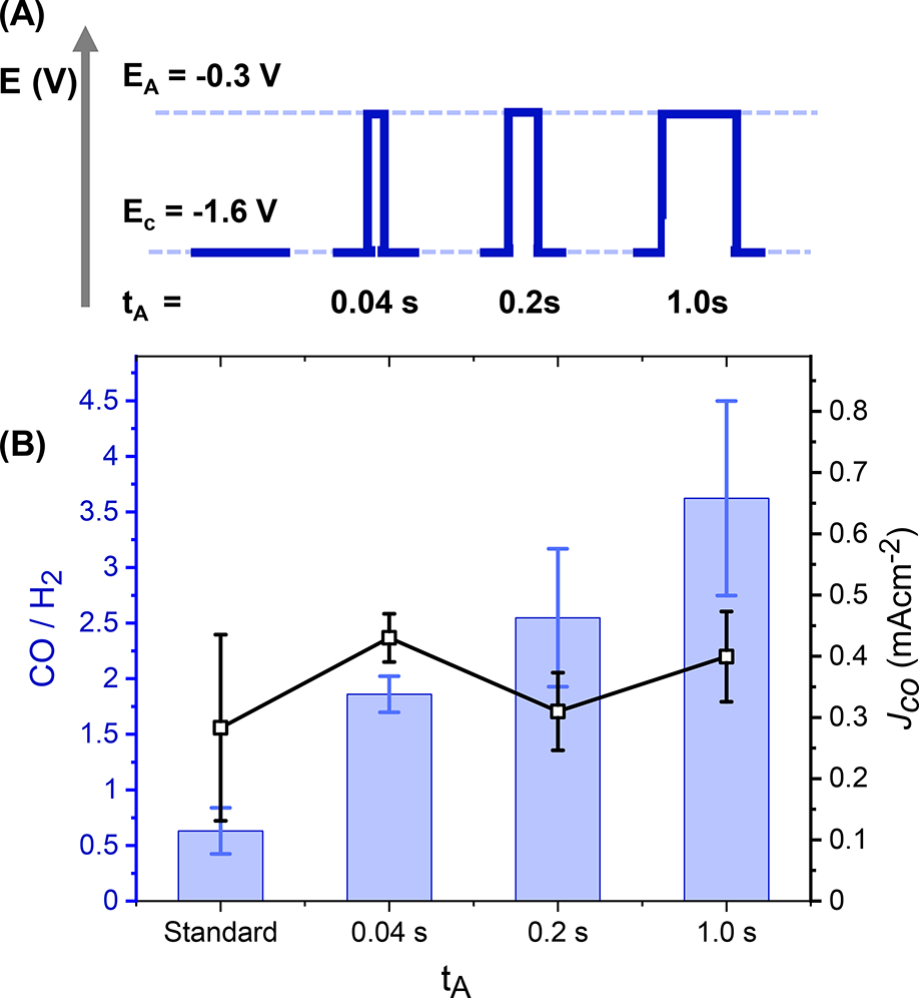
(A) Schematic of different pulse profiles with increasing *t*_a_ (not to scale). (B) Comparison of CO/H_2_ and CO partial current densities after 3 h of electrolysis of 0.1 mM [Ni(cyclam)]^2+^ in 0.5 M NaCl (aq) where *E*_C_ [*t*_C_] = −1.6 V_Ag/AgCl_ [5 s]; *E*_A_= −0.3 V_Ag/AgCl_ with changing *t*_A._.

In conclusion, we here show that short (ms to s) asymmetric voltage pulse profiles can be used to improve the selectivity and achieve stable operation of the molecular catalyst, [Ni(cyclam)]^2+^, for CO_2_R to CO. We find that by rapidly removing [Ni(cyclam)(CO)]^+^, an intermediate on the pathway to an irreversible degradation product, we can achieve a CO/H_2_ selectivity of >1 for up to 12 h without the use of a CO scavenger (Figure S9). We achieve large improvements of activity with anodic pulse durations of just 40 and 200 ms corresponding to duty cycles of >99% and 96%, respectively. More widely, we anticipate the use of short asymmetric pulse profiles may offer a way to modify the activity and stability of a wider range of molecular catalysts through both the in situ regeneration of activated catalytic species and possible non-Faradaic processes.

## Data Availability

All raw data is available at https://doi.org/10.17638/datacat.liverpool.ac.uk/2272.

## Supporting Information Available

Experimental methods, XPS and representative electrolysis data, and extended electrochemical characterization (PDF)

## References

[ref1] Bhugun I.; Lexa D.; Savéant J. M. Catalysis of the Electrochemical Reduction of Carbon Dioxide by Iron(0) Porphyrins: Synergystic Effect of Weak Brönsted Acids. J. Am. Chem. Soc. 1996, 118 (7), 1769–1776. 10.1021/ja9534462.

[ref2] Guo K.; Lei H.; Li X.; Zhang Z.; Wang Y.; Guo H.; Zhang W.; Cao R. Alkali Metal Cation Effects on Electrocatalytic CO2 Reduction with Iron Porphyrins. Chinese Journal of Catalysis 2021, 42 (9), 1439–1444. 10.1016/S1872-2067(20)63762-7.

[ref3] König M.; Vaes J.; Klemm E.; Pant D. Solvents and Supporting Electrolytes in the Electrocatalytic Reduction of CO2. iScience 2019, 19, 135–160. 10.1016/j.isci.2019.07.014. 31369986 PMC6669325

[ref4] Wang Y. Q.; Dan X. H.; Wang X.; Yi Z. Y.; Fu J.; Feng Y. C.; Hu J. S.; Wang D.; Wan L. J. Probing the Synergistic Effects of Mg2+ on CO2 Reduction Reaction on CoPc by in Situ Electrochemical Scanning Tunneling Microscopy. J. Am. Chem. Soc. 2022, 144 (43), 20126–20133. 10.1021/jacs.2c09862. 36259686

[ref5] Casebolt R.; Levine K.; Suntivich J.; Hanrath T. Pulse Check: Potential Opportunities in Pulsed Electrochemical CO2 Reduction. Joule 2021, 5 (8), 1987–2026. 10.1016/j.joule.2021.05.014.

[ref6] Liu T.; Wang J.; Yang X.; Gong M. A Review of Pulse Electrolysis for Efficient Energy Conversion and Chemical Production. Journal of Energy Chemistry 2021, 59, 69–82. 10.1016/j.jechem.2020.10.027.

[ref7] Shiratsuchi R.; Aikoh Y.; Nogami G. Pulsed Electroreduction of CO 2 on Copper Electrodes. J. Electrochem. Soc. 1993, 140 (12), 3479–3482. 10.1149/1.2221113.

[ref8] Lee J.; Tak Y. Electrocatalytic Activity of Cu Electrode in Electroreduction of CO2. Electrochim. Acta 2001, 46 (19), 3015–3022. 10.1016/S0013-4686(01)00527-8.

[ref9] Friebe P.; Bogdanoff P.; Alonso-Vante N.; Tributsch H. A Real-Time Mass Spectroscopy Study of the (Electro)Chemical Factors Affecting CO2 Reduction at Copper. J. Catal. 1997, 168 (2), 374–385. 10.1006/jcat.1997.1606.

[ref10] Lee C. W.; Cho N. H.; Nam K. T.; Hwang Y. J.; Min B. K. Cyclic Two-Step Electrolysis for Stable Electrochemical Conversion of Carbon Dioxide to Formate. Nat. Commun. 2019, 10 (1), 1–8. 10.1038/s41467-019-11903-5. 31477719 PMC6718411

[ref11] Yano J.; Yamasaki S. Pulse-Mode Electrochemical Reduction of Carbon Dioxide Using Copper and Copper Oxide Electrodes for Selective Ethylene Formation. J. Appl. Electrochem. 2008, 38 (12), 1721–1726. 10.1007/s10800-008-9622-3.

[ref12] Jermann B.; Augustynski J. Long-Term Activation of the Copper Cathode in the Course of CO2 Reduction. Electrochim. Acta 1994, 39 (11–12), 1891–1896. 10.1016/0013-4686(94)85181-6.

[ref13] Lin S. C.; Chang C. C.; Chiu S. Y.; Pai H. T.; Liao T. Y.; Hsu C. S.; Chiang W. H.; Tsai M. K.; Chen H. M. Operando Time-Resolved X-Ray Absorption Spectroscopy Reveals the Chemical Nature Enabling Highly Selective CO2 Reduction. Nat. Commun. 2020, 11 (1), 1–12. 10.1038/s41467-020-17231-3. 32665607 PMC7360608

[ref14] Zhang X.-D.; Liu T.; Liu C.; Zheng D.-S.; Huang J.-M.; Liu Q.-W.; Yuan W.-W.; Yin Y.; Huang L.-R.; Xu M.; Li Y.; Gu Z.-Y. Asymmetric Low-Frequency Pulsed Strategy Enables Ultralong CO 2 Reduction Stability and Controllable Product Selectivity. J. Am. Chem. Soc. 2023, 145 (4), 2195–2206. 10.1021/jacs.2c09501. 36629383

[ref15] Lim C. F. C.; Harrington D. A.; Marshall A. T. Altering the Selectivity of Galvanostatic CO2 Reduction on Cu Cathodes by Periodic Cyclic Voltammetry and Potentiostatic Steps. Electrochim. Acta 2016, 222, 133–140. 10.1016/j.electacta.2016.10.185.

[ref16] Lee S. H.; Sullivan I.; Larson D. M.; Liu G.; Toma F. M.; Xiang C.; Drisdell W. S. Correlating Oxidation State and Surface Area to Activity from Operando Studies of Copper CO Electroreduction Catalysts in a Gas-Fed Device. ACS Catal. 2020, 10 (14), 8000–8011. 10.1021/acscatal.0c01670.

[ref17] Casebolt R.; Kimura K. W.; Levine K.; Cimada DaSilva J. A.; Kim J.; Dunbar T. A.; Suntivich J.; Hanrath T. Effect of Electrolyte Composition and Concentration on Pulsed Potential Electrochemical CO2 Reduction. ChemElectroChem. 2021, 8 (4), 681–688. 10.1002/celc.202001445.

[ref18] Oguma T.; Azumi K. Improvement of Electrochemical Reduction of CO2 Using the Potential-Pulse Polarization Method. Electrochemistry 2020, 88 (5), 451–456. 10.5796/electrochemistry.20-00037.

[ref19] Bui J. C.; Kim C.; Weber A. Z.; Bell A. T. Dynamic Boundary Layer Simulation of Pulsed CO2Electrolysis on a Copper Catalyst. ACS Energy Lett. 2021, 6 (4), 1181–1188. 10.1021/acsenergylett.1c00364.

[ref20] Jeon H. S.; Timoshenko J.; Rettenmaier C.; Herzog A.; Yoon A.; Chee S. W.; Oener S.; Hejral U.; Haase F. T.; Cuenya B. R. Selectivity Control of Cu Nanocrystals in a Gas-Fed Flow Cell through CO 2 Pulsed Electroreduction. J. Am. Chem. Soc. 2021, 143, 7578–7587. 10.1021/jacs.1c03443. 33956433 PMC8154520

[ref21] Gupta N.; Gattrell M.; MacDougall B. Calculation for the Cathode Surface Concentrations in the Electrochemical Reduction of CO2 in KHCO3 Solutions. J. Appl. Electrochem. 2006, 36 (2), 161–172. 10.1007/s10800-005-9058-y.

[ref22] Grant J. L.; Goswami K.; Spreer L. O.; Otvos J. W.; Calvin M. Photochemical Reduction of Carbon Dioxide to Carbon Monoxide in Water Using a Nickel(I1) Tetra-Azamacrocycle Complex as Catalyst. J. Chem. Soc., Dalton Trans. 1987, 2105–2109. 10.1039/dt9870002105.

[ref23] Yamazaki Y.; Takeda H.; Ishitani O. Photocatalytic Reduction of CO 2 Using Metal Complexes. Journal of Photochemistry and Photobiology C: Photochemistry Reviews 2015, 25 (4), 106–137. 10.1016/j.jphotochemrev.2015.09.001.

[ref24] Kuehnel M. F.; Sahm C. D.; Neri G.; Lee J. R.; Orchard K. L.; Cowan A. J.; Reisner E. ZnSe Quantum Dots Modified with a Ni(Cyclam) Catalyst for Efficient Visible-Light Driven CO 2 Reduction in Water. Chem. Sci. 2018, 9 (9), 2501–2509. 10.1039/C7SC04429A. 29732127 PMC5911736

[ref25] Fisher B. J.; Eisenberg R. Electrocatalytic Reduction of Carbon Dioxide by Using Macrocycles of Nickel and Cobalt. J. Am. Chem. Soc. 1980, 102 (24), 7361–7363. 10.1021/ja00544a035.

[ref26] Beley M.; Collin J. P.; Ruppert R.; Sauvage J. P. Electrocatalytic Reduction of CO2 by Ni Cyclam2+ in Water: Study of the Factors Affecting the Efficiency and the Selectivity of the Process. J. Am. Chem. Soc. 1986, 108 (24), 7461–7467. 10.1021/ja00284a003. 22283241

[ref27] Beley M.; Collin J.; Ruppert R.; Sauvage J. Nickel(I1)-Cyclam: An Extremely Selective Electrocatalyst for Reduction of C 0 2 in Water. J. Chem. Soc., Chem. Commun. 1984, 2, 1315–1316. 10.1039/c39840001315.

[ref28] Collin J. P.; Jouaiti A.; Sauvage J. P. Electrocatalytic Properties of Ni(Cyclam)2+ and Ni2(Biscyclam)4+ with Respect to CO2and H2O Reduction. Inorg. Chem. 1988, 27 (11), 1986–1990. 10.1021/ic00284a030.

[ref29] Froehlich J. D.; Kubiak C. P. Homogeneous CO_2_ Reduction by Ni(Cyclam) at a Glassy Carbon Electrode. Inorg. Chem. 2012, 51, 3932–3934. 10.1021/ic3001619. 22435533

[ref30] Greenwell F.; Neri G.; Piercy V.; Cowan A. J. Noncovalent Immobilization of a Nickel Cyclam Catalyst on Carbon Electrodes for CO2 Reduction Using Aqueous Electrolyte. Electrochim. Acta 2021, 392, 139015 10.1016/j.electacta.2021.139015.

[ref31] Neri G.; Aldous I. M.; Walsh J. J.; Hardwick L. J.; Cowan A. J. A Highly Active Nickel Electrocatalyst Shows Excellent Selectivity for CO 2 Reduction in Acidic Media †. Chem. Sci. 2016, 7, 1521–1526. 10.1039/C5SC03225C. 28808529 PMC5530941

[ref32] Neri G.; Walsh J. J.; Wilson C.; Reynal A.; Lim J. Y. C.; Li X.; White A. J. P.; Long N. J.; Durrant J. R.; Cowan A. J. A Functionalised Ni Cyclam for CO2 Reduction: Electrocatalysis, Semiconductor Surface Immobilisation and Light-Driven Electron Transfer †. Phys. Chem. Chem. Phys. 2015, 17, 1562–1566. 10.1039/C4CP04871G. 25460350

[ref33] Pugliese S.; Huan N. T.; Solé-Daura A.; Li Y.; Rivera de la Cruz J.-G.; Forte J.; Zanna S.; Krief A.; Su B.-L.; Fontecave M. CO 2 Electroreduction in Water with a Heterogenized C-Substituted Nickel Cyclam Catalyst. Inorg. Chem. 2022, 61 (40), 15841–15852. 10.1021/acs.inorgchem.2c01645. 36166338

[ref34] Pugliese S.; Huan N. T.; Forte J.; Grammatico D.; Zanna S.; Su B.-L.; Li Y.; Fontecave M. Functionalization of Carbon Nanotubes with Nickel Cyclam for the Electrochemical Reduction of CO2. ChemSusChem 2020, 13, 6449–6456. 10.1002/cssc.202002092. 33085837

[ref35] Siritanaratkul B.; Forster M.; Greenwell F.; Sharma P. K.; Yu E. H.; Cowan A. J. Zero-Gap Bipolar Membrane Electrolyzer for Carbon Dioxide Reduction Using Acid-Tolerant Molecular Electrocatalysts. J. Am. Chem. Soc. 2022, 144 (17), 7551–7556. 10.1021/jacs.1c13024. 35451834 PMC9074102

[ref36] Zhanaidarova A.; Moore C. E.; Gembicky M.; Kubiak C. P. Covalent Attachment of [Ni(Alkynyl-Cyclam)] 2+ Catalysts to Glassy Carbon Electrodes. Chem. Commun. 2018, 54 (33), 4116–4119. 10.1039/C8CC00718G. 29620782

[ref37] Froehlich J. D.; Kubiak C. P. The Homogeneous Reduction of CO 2 by [Ni(Cyclam)] + : Increased Catalytic Rates with the Addition of a CO Scavenger. J. Am. Chem. Soc. 2015, 137 (10), 3565–3573. 10.1021/ja512575v. 25714353

[ref38] Balazs G. B.; Anson F. C. Effects of CO on the Electrocatalytic Actiivty of Ni(Cyclam)2+ toward the Reduction of CO2. J. Electroanal. Chem. 1993, 361, 149–157. 10.1016/0022-0728(93)87049-2.

[ref39] Kelly C. A.; Mulazzani Q. G.; Blinn E. L.; Rodgers M. A. J. Kinetics of CO Addition to Ni(Cyclam) + in Aqueous Solution. Inorg. Chem. 1996, 35, 5122–5126. 10.1021/ic951527t.

[ref40] Nichols A. W.; Chatterjee S.; Sabat M.; Machan C. W. Electrocatalytic Reduction of CO2 to Formate by an Iron Schiff Base Complex. Inorg. Chem. 2018, 57 (4), 2111–2121. 10.1021/acs.inorgchem.7b02955. 29384368

[ref41] Cometto C.; Chen L.; Lo P. K.; Guo Z.; Lau K. C.; Anxolabéhère-Mallart E.; Fave C.; Lau T. C.; Robert M. Highly Selective Molecular Catalysts for the CO2-to-CO Electrochemical Conversion at Very Low Overpotential. Contrasting Fe vs Co Quaterpyridine Complexes upon Mechanistic Studies. ACS Catal. 2018, 8 (4), 3411–3417. 10.1021/acscatal.7b04412.

[ref42] Marianov A. N.; Kochubei A. S.; Roman T.; Conquest O. J.; Stampfl C.; Jiang Y. Resolving Deactivation Pathways of Co Porphyrin-Based Electrocatalysts for CO 2 Reduction in Aqueous Medium. ACS Catal. 2021, 11 (6), 3715–3729. 10.1021/acscatal.0c05092.

[ref43] Jiang J.; Matula A. J.; Swierk J. R.; Romano N.; Wu Y.; Batista V. S.; Crabtree R. H.; Lindsey J. S.; Wang H.; Brudvig G. W. Unusual Stability of a Bacteriochlorin Electrocatalyst under Reductive Conditions. A Case Study on CO 2 Conversion to CO. ACS Catal. 2018, 8 (11), 10131–10136. 10.1021/acscatal.8b02991.

[ref44] Wu Y.; Jiang Z.; Lu X.; Liang Y.; Wang H. Domino Electroreduction of CO2 to Methanol on a Molecular Catalyst. Nature 2019, 575 (7784), 639–642. 10.1038/s41586-019-1760-8. 31776492

[ref45] Biesinger M. C.; Payne B. P.; Lau L. W. M.; Gerson A.; Smart R. S. C. X-Ray Photoelectron Spectroscopic Chemical State Quantification of Mixed Nickel Metal, Oxide and Hydroxide Systems. Surf. Interface Anal. 2009, 41 (4), 324–332. 10.1002/sia.3026.

[ref46] Biesinger M. C.; Payne B. P.; Grosvenor A. P.; Lau L. W. M.; Gerson A. R.; Smart R. St. C. Resolving Surface Chemical States in XPS Analysis of First Row Transition Metals, Oxides and Hydroxides: Cr, Mn, Fe, Co and Ni. Appl. Surf. Sci. 2011, 257 (7), 2717–2730. 10.1016/j.apsusc.2010.10.051.

[ref47] McCarthy B. D.; Donley C. L.; Dempsey J. L. Electrode Initiated Proton-Coupled Electron Transfer to Promote Degradation of a Nickel(II) Coordination Complex. Chem. Sci. 2015, 6 (5), 2827–2834. 10.1039/C5SC00476D. 29403633 PMC5761499

[ref48] Randin J.-P.; Yeager E. Differential Capacitance Study on the Edge Orientation of Pyrolytic Graphite and Glassy Carbon Electrodes. J. Electroanal Chem. Interfacial Electrochem 1975, 58 (2), 313–322. 10.1016/S0022-0728(75)80089-1.

[ref49] Zebardast H. R.; Rogak S.; Asselin E. Potential of Zero Charge of Glassy Carbon at Elevated Temperatures. J. Electroanal. Chem. 2014, 724, 36–42. 10.1016/j.jelechem.2014.03.030.

