# Supplementary material for: Pulsed Electrolysis with a Nickel Molecular Catalyst Improves Selectivity for Carbon Dioxide Reduction
Source: J Am Chem Soc. 2023 Jul 5;145(28):15078–83. doi: 10.1021/jacs.3c04811 (PMC10360055; doi:10.1021/jacs.3c04811)
Supplement: Supplementary file 1 — ja3c04811_si_001.pdf [file ja3c04811_si_001.pdf]

Supplementary Information

Pulsed Electrolysis with a Nickel Molecular Catalyst Improves Selectivity for Carbon Dioxide Reduction

Francesca Greenwell[a], Bhavin Siritanaratkul[a], Preetam K. Sharma[b], Eileen H. Yu[b] and Alexander J. Cowan[a]*

[a]Department of Chemistry and Stephenson Institute for Renewable Energy, University of Liverpool, Liverpool L69 7ZF, United Kingdom

[b]Department of Chemical Engineering, Loughborough University, Loughborough LE11 3TU, United Kingdom

Experimental Methods

Synthesis of [Ni(Cyc)]Cl_2

Cyclam ligand (1,4,8,11- tetraazacyclotetradecane) (607 mg, 3.01 mmol) was added to EtOH (100 mL) and stirred until completely dissolved. $NiCl_2$•$6H_2O$ (720 mg, 3.03 mmol) was added to the solution, and left stirring overnight at room temperature. Diethyl ether was added to precipitate the [Ni(Cyc)]Cl_2, and the precipitates were filtered, collected and dried in air. Obtained: 817 mg, yield: 82%. MS (ESI+) 257 [M+ -2Cl]; CHN microanalysis: Calculated for C10H24Cl2N4Ni: C, 36.40; H, 7.33; N, 16.98. Found: C, 36.39; H, 7.31; N, 16.92.

Electrochemistry

Electrochemical measurements were carried out using Biologic SP-200, Biologic VSP and Ivium Vertex potentiostats. All electrochemistry was run in a standard glass half-cell using a glassy carbon electrode (A = 0.071 cm^2) (IJ Cambria Scientific Ltd) as the working electrode, a platinum mesh as the counter electrode, separated by a glass sleeve with a Vycor frit and a Ag/AgCl reference electrode (see picture below). The GC working electrode was polished with 1.0 µm and 0.05 µhm Micropolish™ Alumina on 8” mircocloth (Buehler) for 4 minutes before sonicating with Milli-Q. Electrolyte was 0.5 M NaCl from Merck (99.5%) in Milli-Q water (18.2 MΩ) pre-electrolysed (-0.1mA) overnight with a titanium plate (working) and carbon counter.[1] The cell was purged with either N_2, CO_2 (for cyclic voltammetry and impedance spectroscopy) or CO_2 with 1% CH_4 (BOC) (for chronoamperometry) for ~30 minutes prior to experiments. All electrochemistry was run at room temperature and pressure. Electrolysis experiments used the same cell and electrode configuration as the CV studies (image below) with the addition of a magnetic stirrer bar. The experiments were run in triplicate, with total current densities and GC measured selectivity the average of all runs with errors calculated as one standard deviation. The error bars given for values derived from these measurements (e.g. partial current densities) were obtained through standard error propagation methods. The current sampling-rate in the long-pulsed electrolysis experiments becomes limited by the large number of data points generated and the intrinsic limitations of the software used (EC lab). For a more detailed kinetic analysis of the chronoamperometric response we carried data collection over a short time period (50 s) with a high current sampling rate (sub-ms data resolution, figure S8). These short, high sampling-rate, data

collection periods took place immediately after a period of electrolysis (30 minutes) using the standard data sampling rate.

Product detection

Gaseous products were measured by gas chromatography, by taking manual injections directly from the cell headspace and analysed using an Agilent 6890N with a 5 A molecular sieve column (ValcoPLOT, 30 m length, 0.53 mm ID) and a pulsed discharge detector (D-3-I-HP, Valco Vici). Moles of product were quantified using a calibration curve from known concentrations of H_2, CO and CH_4. A CH_4 internal calibrant of known concentration was also used in the cell to confirm the accuracy of the calibration

$$Faradaic\ efficiency(\%) = \frac{moles(products) \times no.(electrons\ for\ reaction)}{moles(electrons)} \times 100$$

The faradaic efficiency was calculated using the equation above where the number of electrons for the reaction is 2. Moles of electrons are obtained from charge passed during electrolysis.

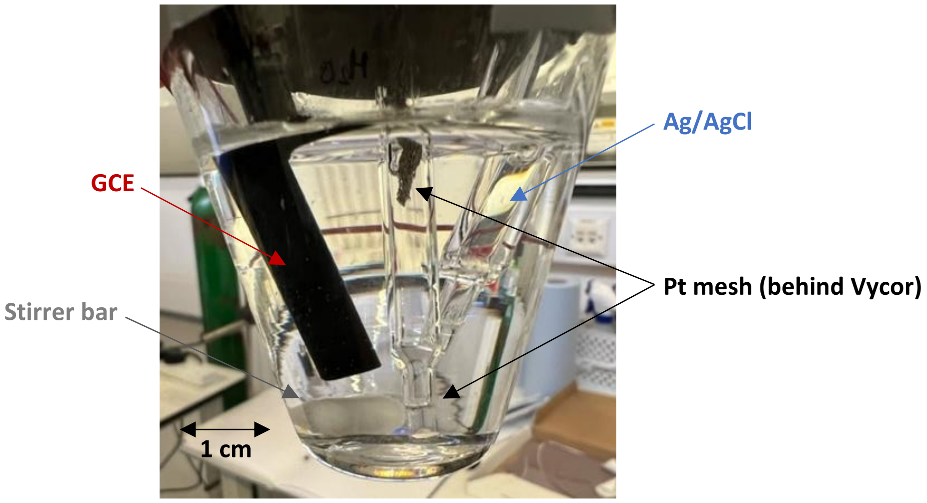

Figure S1 *Photograph of cell set-up used in electrolysis*

X-ray photonelectron spectroscopy (XPS)

X-ray photoelectron spectroscopy (XPS) was utilised to determine the nature of deposited species on the glassy carbon surface. The samples underwent 3 h standard and pulsed (E_A = -1.0 V) electrolysis in 0.1 mM Ni(cyclam) in 0.5 M NaCl and wee thoroughly washed with Milli-Q water and dried with compressed air before inserting in the XPS chamber. Analysis of a powder sample of Ni(cyclam) was used for comparison and is reproduced from Siritanaratkul with permission.[2]

The measurements were performed on a Kratos Axis Supra instrument using Al K_α X-ray source. The survey scans were performed at 80 eV pass energy and high-resolution scans were performed at 20 eV pass energy. The energy calibration was performed using O 1s peak at 530.9 eV.

Double Potential Step Chronocoulometry (DPSC) of Ni(cyclam) on GCE

Here double potential step chronocoulometry was performed on 0.1 mM Ni(cyclam) to assess the adsorption of catalyst onto GC working electrode. This was done using methods from literature,[3,4] where the potential of interest was held for 30 s to allow reductive adsorption of $[Ni(cyclam)]^+$ to occur, the potential was then jumped to +0.2 V_{NHE} in the first step and then stepped back to the initial potential. The charge passed over 10 ms in the first step was plotted *vs* $t^{1/2}$ while the charge passed over 10 ms in the second step was plotted *vs* $[\tau^{1/2} + (t-\tau)^{1/2} - t^{1/2}]$ where τ is the duration of the first step and t is the time. The difference of the forward and reverse intercepts gives charge of adsorbed species, Q_{ads}, from which the surface coverage, Γ, was calculated using: $\Gamma = \frac{Q_{ads}}{FnA}$ where F the Faraday constant, n the number of electrons and A the electrode area in cm^2.

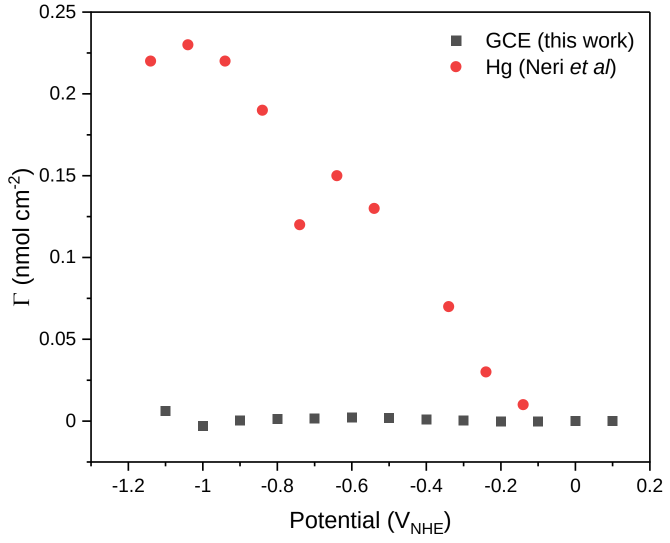

Figure S2. *Dependence of the surface coverage of 0.1 mM Ni(cyclam) on GCE in 0.5 M NaCl (black square) compared with 0.1 mM Ni(cyclam) on Hg drop in 0.1 M $NaClO_4$, pH 2 (red circle). Hg data reproduced from Neri et al with permission.*[4]

Faradaic efficiencies

Table S1 *Bulk electrolysis data after 1 h*

Sample	E_C (V) [t_c]	E_A (V) [t_a]	J (mAcm^{-2})	FE_{H2} (%)	FE_{CO} (%)	FE_{Total} (%)
Standard	-1.60	NA	0.59±0.26	46.7±13.75	43.66±9.24	90.36±16.56
Pulse	-1.60 [5 s]	-0.3 [0.2 s]	0.43±0.03	23.94±3.10	67.3±15.18	91.25±15.50
Pulse	-1.60 [5 s]	-0.8 [0.2 s]	0.54±0.17	21.43±5.25	58.05±2.50	79.48±13.03
Pulse	-1.60 [5 s]	-1.0 [0.2 s]	0.62±0.03	20.66±9.49	52.88±11.93	73.53±15.24
Pulse	-1.60 [5 s]	-1.3 [0.2 s]	0.58±0.19	39.49±2.03	39.52±9.91	79.01±10.11
Pulse	-1.60 [5 s]	-0.3 [0.04 s]	0.66±0.17	38.38±8.73	68.31±7.99	106.69±11.83
Pulse	-1.60 [5 s]	-0.3 [1 s]	0.45±0.14	15.24±9.62	79.08±14.01	94.32±17.00

Table S2 *Bulk electrolysis data after 2 h*

Sample	E_C (V) [t_c]	E_A (V) [t_a]	J (mAcm^{-2})	FE_{H2} (%)	FE_{CO} (%)	FE_{Total} (%)
Standard	-1.60	NA	0.74±0.31	51.89±11.71	39.22±11.69	91.11±16.55
Pulse	-1.60 [5 s]	-0.3 [0.2 s]	0.43±0.03	25.68±4.54	73.03±15.56	98.72±16.21
Pulse	-1.60 [5 s]	-0.8 [0.2 s]	0.60±0.16	23.88±4.54	59.77±1.07	83.66±3.63
Pulse	-1.60 [5 s]	-1.0 [0.2 s]	0.66±0.05	23.68±3.47	63.06±5.16	86.74±6.16
Pulse	-1.60 [5 s]	-1.3 [0.2 s]	0.75±0.19	45.97±11.63	35.98±1.62	81.95±11.75
Pulse	-1.60 [5 s]	-0.3 [0.04 s]	0.68±0.14	33.54±6.84	65.14±3.74	98.70±7.79
Pulse	-1.60 [5 s]	-0.3 [1 s]	0.43±0.06	21.06±6.23	84.60±13.19	105.66±14.59

Table S3 *Bulk electrolysis data after 3h*

Sample	E_C (V) [t_c]	E_A (V) [t_a]	J ($mAcm^{-2}$)	FE_{H2} (%)	FE_{CO} (%)	FE_{Total} (%)
Standard	-1.60	NA	0.81±0.40	55.56±10.37	35.12±10.31	90.68±14.63
Pulse	-1.60 [5 s]	-0.3 [0.2 s]	0.43±0.01	28.57±5.61	72.81±14.90	101.39±15.92
Pulse	-1.60 [5 s]	-0.8 [0.2 s]	0.69±0.30	29.17±5.80	60.19±6.65	89.35±8.83
Pulse	-1.60 [5 s]	-1.0 [0.2 s]	0.69±0.11	26.76±3.10	64.86±1.91	91.61±3.62
Pulse	-1.60 [5 s]	-1.3 [0.2 s]	0.79±0.20	52.29±16.71	36.13±6.93	88.42±18.10
Pulse	-1.60 [5 s]	-0.3 [0.04 s]	0.70±0.13	33.04±5.92	61.47±3.38	94.51±6.82
Pulse	-1.60 [5 s]	-0.3 [1 s]	0.46±0.05	23.62±6.17	85.55±14.80	109.17±16.03

Electrolysis Traces

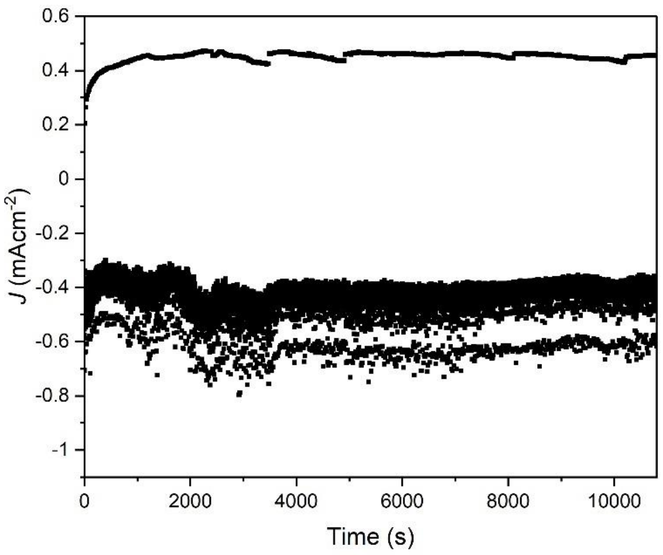

Figure S3 *Chronoamperometry trace of a typical pulsed run of 0.1 mM Ni(cyclam) in 0.5 M NaCl (aq) where E_A = -0.3 $V_{Ag/AgCl}$ and t_A = 0.2 s. Data for all repeats can be found in the data catalogue at* https://doi.org/10.17638/datacat.liverpool.ac.uk/2272

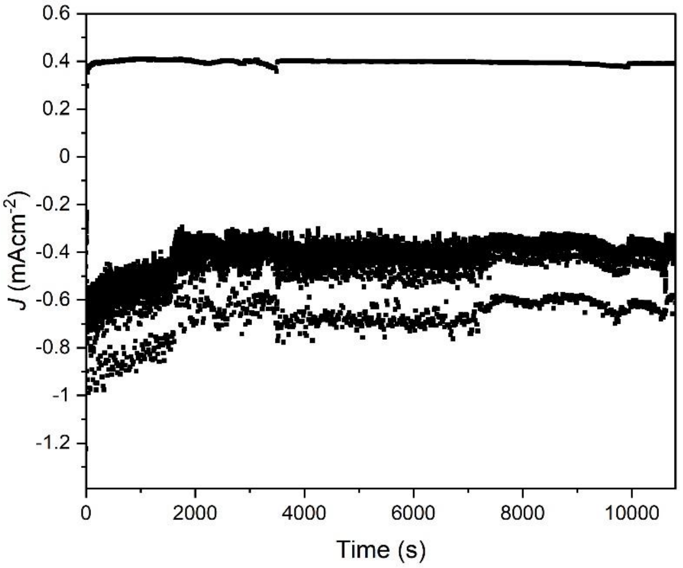

Figure S4 *Chronoamperometry trace of pulsed run of 0.1 mM Ni(cyclam) in 0.5 M NaCl (aq) where E_A = -0.8 $V_{Ag/AgCl}$ and t_A = 0.2 s. Data for all repeats can be found in the data catalogue at* https://doi.org/10.17638/datacat.liverpool.ac.uk/2272

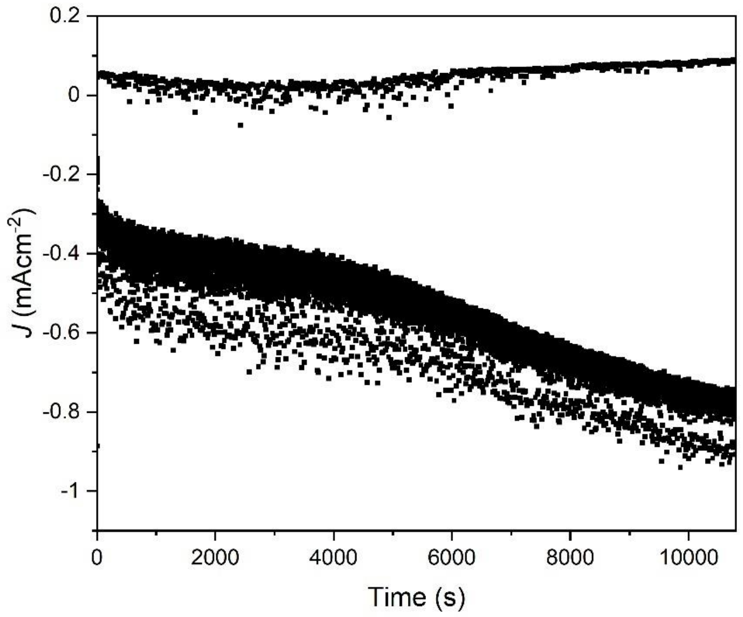

Figure S5 *Chronoamperometry trace of pulsed run of 0.1 mM Ni(cyclam) in 0.5 M NaCl (aq) where E_A = -1.3 $V_{Ag/AgCl}$ and t_A = 0.2 s. Data for all repeats can be found in the data catalogue at* https://doi.org/10.17638/datacat.liverpool.ac.uk/2272

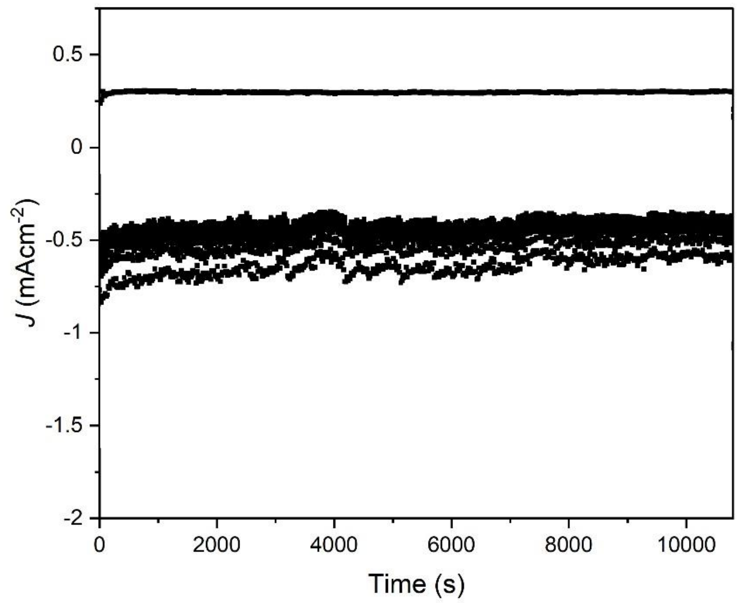

Figure S6 *Chronoamperometry trace of pulsed run of 0.1 mM Ni(cyclam) in 0.5 M NaCl (aq) where E_A = -0.3 $V_{Ag/AgCl}$ and t_A = 1.0 s Data for all repeats can be found in the data catalogue at* https://doi.org/10.17638/datacat.liverpool.ac.uk/2272

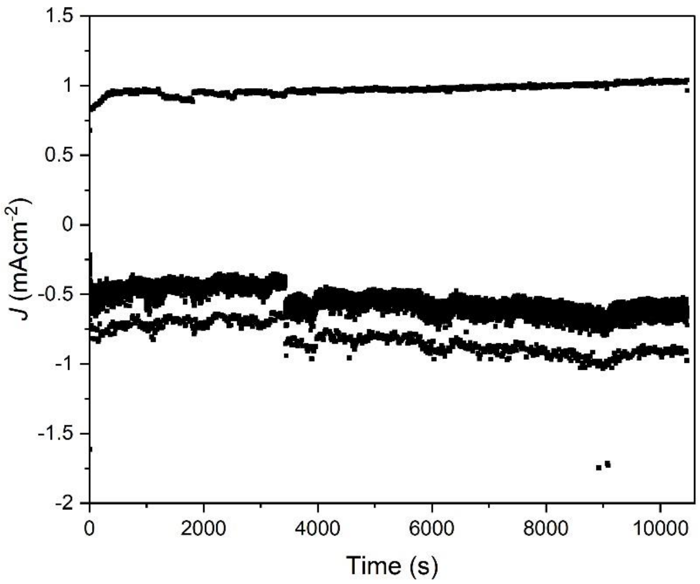

Figure S7 *Chronoamperometry trace of pulsed run of 0.1 mM Ni(cyclam) in 0.5 M NaCl (aq) where E_A = -0.3 $V_{Ag/AgCl}$ and t_A = 0.04 s. Data for all repeats can be found in the data catalogue at* https://doi.org/10.17638/datacat.liverpool.ac.uk/2272

Calculation of Full Cell Voltage Efficiency (VE) and Full Cell Energy Efficiency (EE)

$$Full\ Cell\ Voltage\ Efficiency: \quad VE_{full\ cell} = \frac{1.23 + (-E_{CO})}{(E_{full\ cell})}$$

$$CO\ Full\ Cell\ Energetic\ Efficiency: \quad EE_{full\ cell} = VE_{full\ cell} \times FE_{CO}$$

Where $E_{full\ cell}$ is the full cell applied potential; E_{CO} = -0.109 *V* is thermodynamic potential (*vs* RHE) of CO_2 reduction to CO and FE_{CO} is the measured CO Faradaic efficiency as a percentage.[5]

Table S4. *Full cell energy efficiencies of standard and pulse electrolysis of 0.1 mM NiCyc and 0.5 M NaCl*

Sample	Av. E_{WE} (V)	Av. E_{CE} (V)	$E_{full\ cell}$ (V)	$VE_{full\ cell}$	FE_{CO} (%)	$EE_{full\ cell}$ (%)
Standard	-1.60	1.32	2.92	0.46	35.12	**16.16**
Pulse (Ea = -1.0V)	-1.58	1.56	3.14	0.43	64.86	**27.89**

Pulse profile: Analysis of Faradaic and non-Faradaic contributions

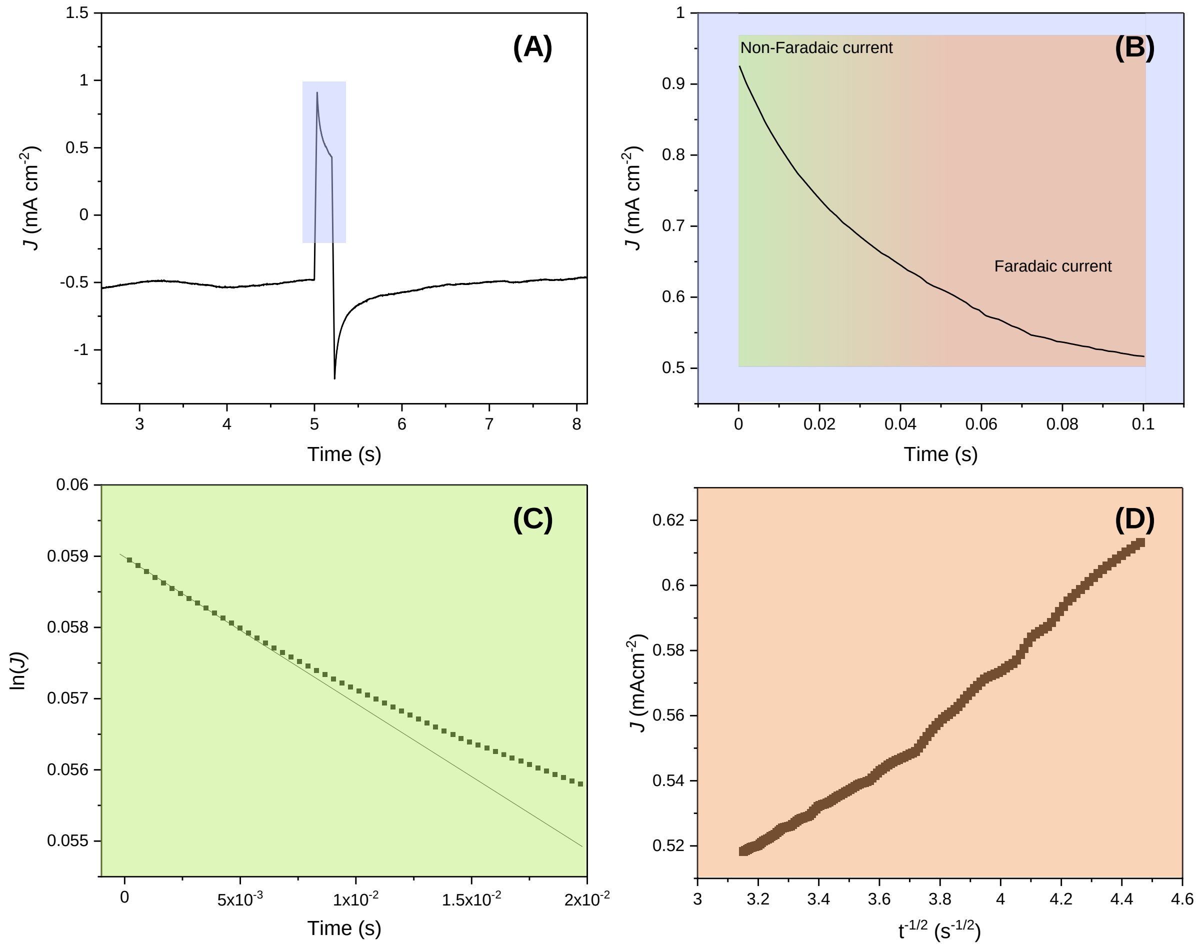

Figure S8 *(A) Full pulse profile (ta = 200 ms; Ea = -0.3 V vs Ag/AgCl; tc = 5 s; Ec = -1.6 V vs Ag/AgCl). (B) Total anodic current averaged over 10 pulses. (C) Natural log of anodic current showing non-faradaic current in the linear regime. (D) Faradaic current plotted against* $t^{-1/2}$.

Here the cell was pulsed 10 times (< 1 min) at a higher time resolution in order to break down the anodic pulse profile (Ea = -0.3$V_{Ag/AgCl}$) into faradaic and non-faradaic regions using methods done by Kimura *et al.*[10] In Fig S8(c) the natural log of current density is plotted against time, here we see deviation from the linear trend after ~10 ms, suggesting that Faradaic current begins to contribute significantly past this time.

Comparison of standard and pulse electrolysis during extended experiments

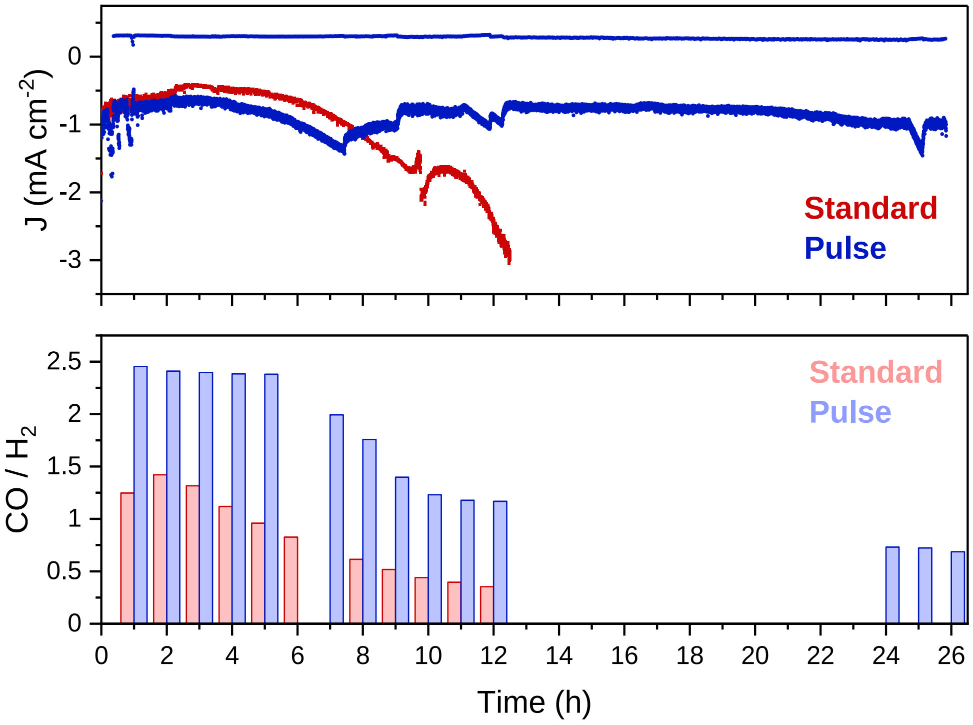

Figure S9 *Selectivity's (columns) and current densities (symbol) of standard and pulse electro lysis of 0.1 mM Ni(cyclam) in 0.5 M NaCl (aq) over 12 to 26 h. Standard electrolysis (E = -1.6 V) (red) and pulse electrolysis (Ec = -1.6 V (5s) Ea = -1.0 V (0.2 s)) (blue). The standard experiment was stopped after 12 hours due to the rapid increase in current due to increased hydrogen evolution.*

X-ray photoelectron spectroscopy (XPS)

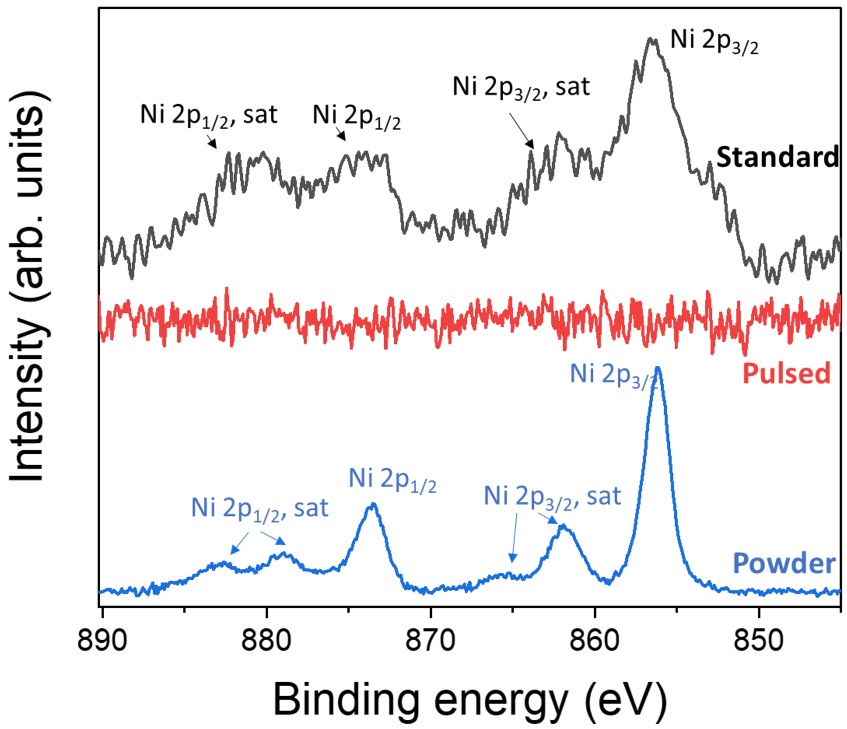

Figure S10 *Ni 2p XPS spectra for glassy carbon substrate post 3 h standard (black) and pulse electrolysis (red). The spectrum of Ni(cyclam) powder (blue) is added at the bottom for reference.*

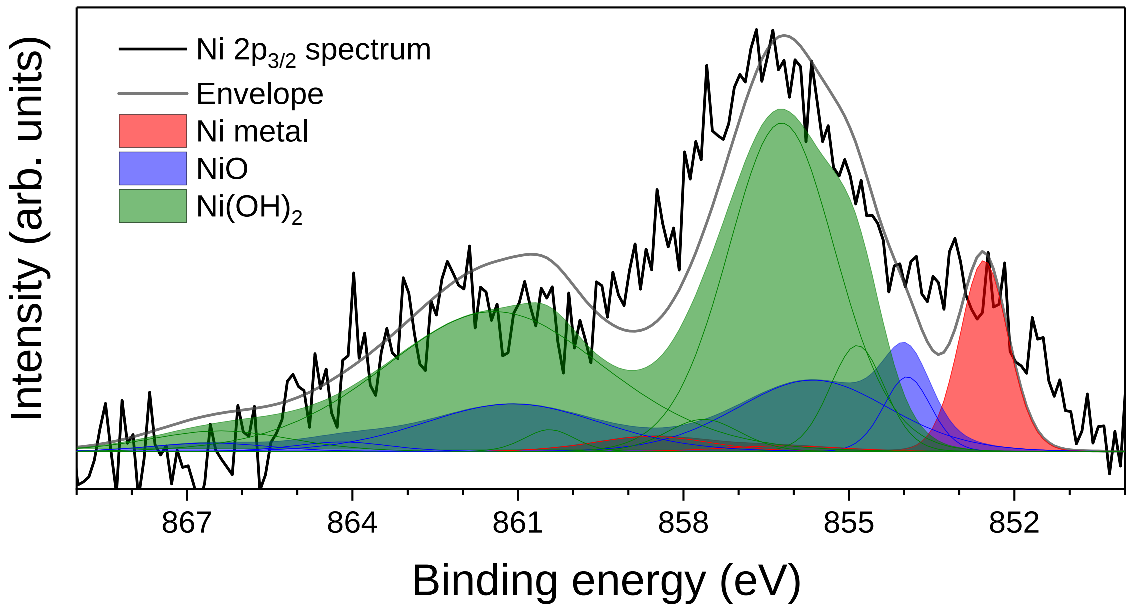

Figure S11 *Deconvolution of Ni 2p XPS spectra of glassy carbon substrate post 3 h standard electrolysis showing peaks corresponding to Ni, NiO and Ni(OH)$_2$*

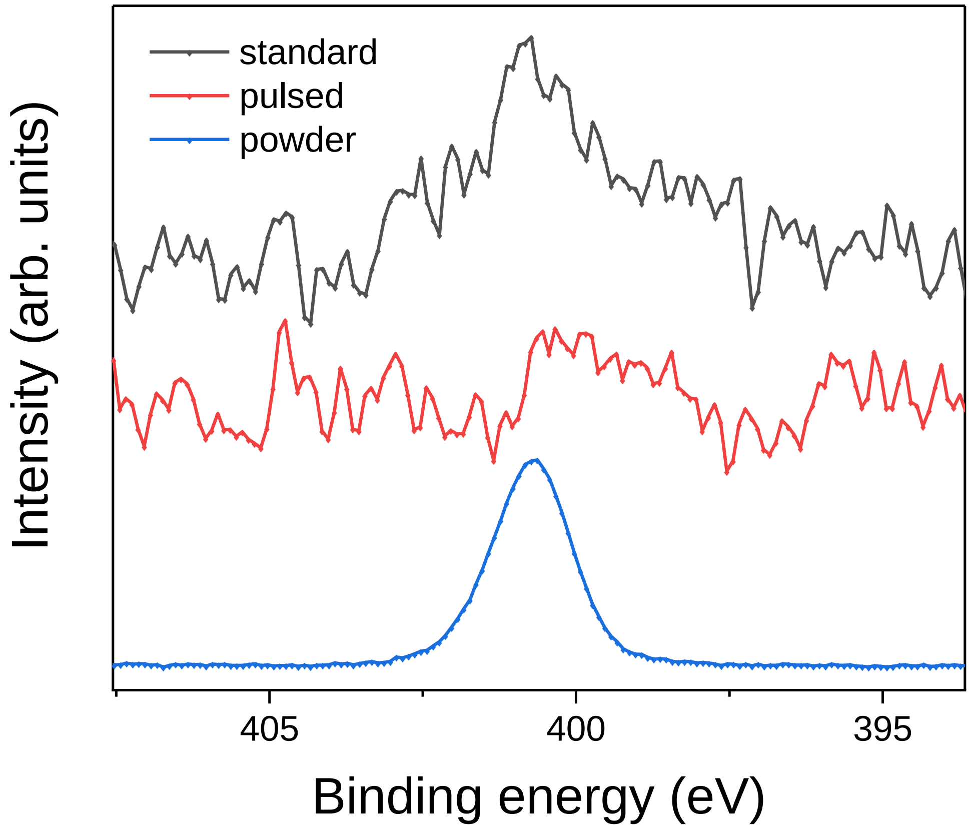

Figure S12 *N 1s XPS spectra for glassy carbon substrate post 3 h standard (black) and pulse electrolysis (red). The spectrum of Ni(cyclam) powder (blue) is added at the bottom for reference.*

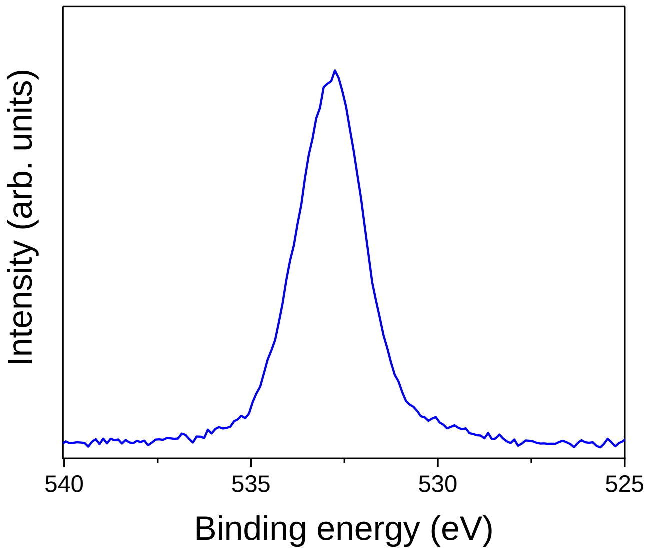

Figure S13 *O 1s spectrum for glassy carbon substrate post 3h standard electrolysis*

As shown in the Fig S10, the spectrum for the standard sample is different from the Ni(cyclam) powder indicating a change in structure. The limited amount of Ni on the surface resulted in noisy spectrum, which was deconvoluted using literature parameters for Ni, NiO and $Ni(OH)_2$ structures and is shown in Fig S11. In the spectrum, multiplet splitting arises due to the presence of unpaired electrons which upon interaction with the core electrons, can create multiple energy levels.[6] By the deconvolution, it was determined that $Ni(OH)_2$ is the dominating species with 68.5% composition on the surface.[7] With the remainder in NiO (21.5%) and Ni metal forms. O 1s spectrum was analysed for further insight into Ni form remaining on the standard sample (Fig S13). The presence of single peak of O 1s further corroborates that Ni hydroxide is the dominating phase.[8]

For the pulsed sample (red, Fig S3), there is no Ni of any form left on the surface. The absence of corresponding N 1s peak in figure 2, confirms the absence of Ni(cyclam) from the surface.

CVs of clean and fouled electrode

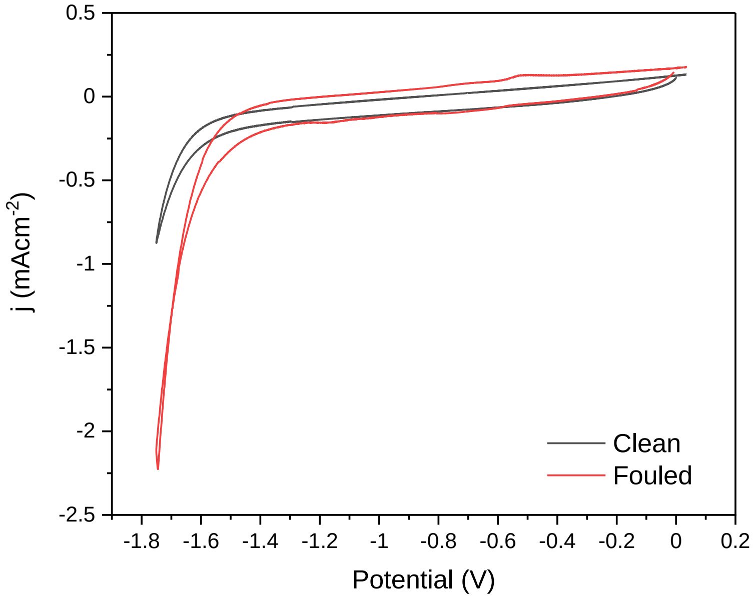

Fig S14 *CVs of GCE in 0.5 M NaCl(aq) under Ar both before (Clean) and after (Fouled) 3 h standard electrolysis in 0.1 mM Ni(cyclam) in 0.5 M NaCl(aq) at -1.6* $V_{Ag/AgCl}$

Differential capacitance curves for calculating PZC

The impedance of the system can be modelled as an ohmic resistance and a capacitance (the double layer capacitance of the glassy carbon electrode) in series, as follows:

$$Differential\ Capacitance\ from\ measured\ impedance:$$

$$Z = Z_{Re} + iZ_{Im} = R + (iC\omega)^{-1}$$

$$C = -(\omega Z_{Im})^{-1}\ where\ \ \omega = 2\pi f$$

Where Z is the impedance of the interfacial region between the working and reference electrode, where Z_{Re} and Z_{Im} are the real and imaginary parts respectively; ω is the angular frequency of the ac perturbation; f is the frequency. Here the capacitance is calculated using the single-frequency impedance over a potential range of +0.6 to -1.2 $V_{Ag/AgCl}$ recording every 36 mV with 1-minute equilibration time between each potential with an amplitude (V_a) of 10 mV using methods from literature.[9]

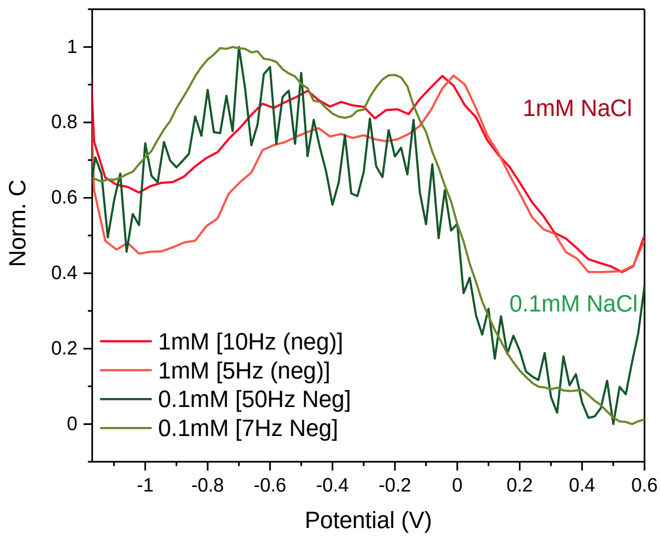

Figure S15 *Normalised differential capacitance curves of GCE in NaCl (aq)*

Charge ratios with changing pulse parameters

$$Q_{total} = q_C + q_A$$

$$Q_C\,(\%) = \frac{q_C}{Q_{total}} \times 100 \qquad Q_A\,(\%) = \frac{q_A}{Q_{total}} \times 100$$

Where q_C is all cathodic charge passed, q_A is all anodic charge passed and Q_{total} is all charge passed.

$$Duty\ Cycle\,(\%) = \frac{t_C}{T} \times 100$$

Where t_c is time spent at the cathodic potential (undergoing eCO_2R) and T is total time of cycle.

**E_C (V) [t_C]**	**E_A (V) [t_A]**	**Q_C (%)**	**Q_A (%)**	**Duty Cycle (%)**
-1.6 [5.0s]	-0.3 [0.2 s]	96.3	3.7	96.2
	**-0.8 [0.2 s]**	**97.9**	**2.1**	**96.2**
	**-1.0 [0.2 s]**	**98.7**	**1.3**	**96.2**
	**-0.3 [0.04 s]**	**97.5**	**2.5**	**99.2**
	-0.3 [1.0 s]	91.2	8.8	83.3

Table S5 *The effective charge in both the cathodic and anodic regions*

This highlights the benefit of applying a less positive E_a and shorter t_a with a of $Q_c > 97$ % for methods highlighted in bold.[11]

References

(1) Hori, Y.; Konishi, H.; Futamura, T.; Murata, A.; Koga, O.; Sakurai, H.; Oguma, K. "Deactivation of Copper Electrode" in Electrochemical Reduction of CO2. *Electrochim. Acta* **2005**, *50* (27), 5354–5369. https://doi.org/10.1016/j.electacta.2005.03.015.

(2) Siritanaratkul, B.; Forster, M.; Greenwell, F.; Sharma, P. K.; Yu, E. H.; Cowan, A. J. Zero-Gap Bipolar Membrane Electrolyzer for Carbon Dioxide Reduction Using Acid-Tolerant Molecular Electrocatalysts. *J. Am. Chem. Soc.* **2022**, *144* (17), 7551–7556. https://doi.org/10.1021/jacs.1c13024.

(3) Balazs, G. B.; Anson, F. C. The Adsorption of Ni(Cyclam)+ at Mercury Electrodes and Its Relation to the Electrocatalytic Reduction of CO2. *J. Electroanal. Chem.* **1992**, *322* (1–2), 325–345. https://doi.org/10.1016/0022-0728(92)80086-J.

(4) Neri, G.; Aldous, I. M.; Walsh, J. J.; Hardwick, L. J.; Cowan, A. J. Chemical Science A Highly Active Nickel Electrocatalyst Shows Excellent Selectivity for CO 2 Reduction in Acidic Media †. *Chem. Sci.* **2016**, *7*, 1521–1526. https://doi.org/10.1039/c5sc03225c.

(5) Gabardo, C. M.; Seifitokaldani, A.; Edwards, J. P.; Dinh, C. T.; Burdyny, T.; Kibria, M. G.; O'Brien, C. P.; Sargent, E. H.; Sinton, D. Combined High Alkalinity and Pressurization Enable Efficient CO2 Electroreduction to CO. *Energy Environ. Sci.* **2018**, *11* (9), 2531–2539. https://doi.org/10.1039/c8ee01684d.

(6) Moulder, J. F.; Stickle, W. F.; Sobol, P. E.; Bomben, K. D. *Handbook of X-Ray Photoelectron Spectroscopy*; Chastain, J., Ed.; Perkin-Elemer Corp. 1992. https://doi.org/10.1002/0470014229.ch22.

(7) Biesinger, M. C.; Payne, B. P.; Grosvenor, A. P.; Lau, L. W. M.; Gerson, A. R.; Smart, R. St. C. Resolving Surface Chemical States in XPS Analysis of First Row Transition Metals, Oxides and Hydroxides: Cr, Mn, Fe, Co and Ni. *Appl Surf Sci* **2011**, *257* (7), 2717–2730. https://doi.org/10.1016/j.apsusc.2010.10.051.

(8) Biesinger, M. C.; Payne, B. P.; Lau, L. W. M.; Gerson, A.; Smart, R. S. C. X-Ray Photoelectron Spectroscopic Chemical State Quantification of Mixed Nickel Metal, Oxide and Hydroxide Systems. *Surface and Interface Analysis* **2009**, *41* (4), 324–332. https://doi.org/10.1002/sia.3026.

(9) Lockett, V.; Sedev, R.; Ralston, J.; Horne, M.; Rodopoulos, T. Differential Capacitance of the Electrical Double Layer in Imidazolium-Based Ionic Liquids: Influence of Potential, Cation Size, and Temperature. *J. Phys. Chem. C* **2008**, *112* (19), 7486–7495. https://doi.org/10.1021/jp7100732.

(10) Kimura, K. W.; Fritz, K. E.; Kim, J.; Suntivich, J.; Abruña, H. D.; Hanrath, T. Controlled Selectivity of CO 2 Reduction on Copper by Pulsing the Electrochemical Potential. *ChemSusChem* **2018**, *11* (11), 1781–1786. https://doi.org/10.1002/cssc.201800318.

(11) Zhang, X.-D.; Liu, T.; Liu, C.; Zheng, D.-S.; Huang, J.-M.; Liu, Q.-W.; Yuan, W.-W.; Yin, Y.; Huang, L.-R.; Xu, M.; Li, Y.; Gu, Z.-Y. Asymmetric Low-Frequency Pulsed Strategy Enables Ultralong CO 2 Reduction Stability and Controllable Product Selectivity. *J Am Chem Soc* **2023**, *145* (4), 2195–2206. https://doi.org/10.1021/jacs.2c09501.
